# Smart Search System of Autonomous Flight UAVs for Disaster Rescue

**DOI:** 10.3390/s21206810

**Published:** 2021-10-13

**Authors:** Donggeun Oh, Junghee Han

**Affiliations:** 1 Hyundai Autoever, Teheran-Ro 114, Seoul 06176, Korea; gon023415@kau.kr; 2 School of Electronics and Information Engineering, Korea Aerospace University, 76 Hanggongdaehang-ro, Goyang-si 10540, Korea

**Keywords:** UAV (Unmanned Aerial Vehicles), disaster, localization, smart search, autonomous flight

## Abstract

UAVs (Unmanned Aerial Vehicles) have been developed and adopted for various fields including military, IT, agriculture, construction, and so on. In particular, UAVs are being heavily used in the field of disaster relief thanks to the fact that UAVs are becoming smaller and more intelligent. Search for a person in a disaster site can be difficult if the mobile communication network is not available, and if the person is in the GPS shadow area. Recently, the search for survivors using unmanned aerial vehicles has been studied, but there are several problems as the search is mainly using images taken with cameras (including thermal imaging cameras). For example, it is difficult to distinguish a distressed person from a long distance especially in the presence of cover. Considering these challenges, we proposed an autonomous UAV smart search system that can complete their missions without interference in search and tracking of castaways even in disaster areas where communication with base stations is likely to be lost. To achieve this goal, we first make UAVs perform autonomous flight with locating and approaching the distressed people without the help of the ground control server (GCS). Second, to locate a survivor accurately, we developed a genetic-based localization algorithm by detecting changes in the signal strength between distress and drones inside the search system. Specifically, we modeled our target platform with a genetic algorithm and we re-defined the genetic algorithm customized to the disaster site’s environment for tracking accuracy. Finally, we verified the proposed search system in several real-world sites and found that it successfully located targets with autonomous flight.

## 1. Introduction

It is trivial that if the survivors in a disaster site can find out their location using GPS, then we can easily determine the survivor’s location. However, it might become very difficult to request a rescue if the mobile communication network is not reachable or the exact location of the person is not available because the person is in the GPS shadow area.

Recently, the search for survivors using unmanned aerial vehicles (UAVs) has been attempted in many cases [1,2,3]. These platforms mainly use images taken with regular cameras and/or thermal imaging cameras. Since regular cameras have inherent limitations, thermal cameras have often been used together so that people can be found regardless of day or night, and it is easy to distinguish them. However, they are expensive; as such, it must be mounted on an expensive drone, so the overall drone price is inevitably very expensive. Furthermore, there are several challenges in use of either thermal imaging cameras or general cameras. First, it is difficult to identify objects from a distance, so UAVs with cameras have to be taken close up. As a consequence, it takes a lot of time to search a large area. At this time, one flight time of the rotary wing drone is 2 to 30 min, so you have to keep returning and replacing the battery while searching. Furthermore, in the presence of cover, it becomes more difficult to distinguish a distressed person.

On the other hand, if you use a drone to detect radio waves and search the location of the survivor, the time required for the initial search can be drastically reduced and a large area can be searched quickly. In addition to mobile communication radio waves, by detecting radio waves from WiFi and portable RF terminals, location search becomes possible even in non-cellular areas or in GPS shadow areas.

From this point of view, the main purpose of the system proposed in this paper is to expedite the search operation in the disaster areas outside the mobile communication network where distressed persons cannot request rescue. The speed of rescue is directly related to the lives of the survivors, and a quick search operation can minimize cost. To achieve this goal, we develop a smart search system consisting of autonomous flying UAV, GCS, a smart search algorithm, and protocols. This system allows UAVs to locate and approach people in distress and perform autonomous flight without direct control of ground control servers.

The main contributions of the proposed paper can be summarized as follows:

- Autonomous UAV Search: the smart search system enables UAVs to perform the autonomous search process to locate and approach the distressed people without the help of the ground control server (GCS). When a UAV takes off, the first predicted position is not accurate and can be very far from the actual survivor’s position. As the drone flies, it accumulates RSSI (Received Signal Strength Indicator) and ToA (Time of Arrival) data from survivors and the UAV gradually modifies its flight direction towards the survivor, resulting in a more accurate estimate of the location.
- Quick and Smart Tracking Algorithm: we present a smart search system based on a genetic algorithm by detecting changes in the signal strength between distress and drones inside the search system. The proposed smart search system is customized to the disaster site environment to improve tracking accuracy. Specifically, by combining RSSI and ToA data in consideration of the flight environment, it is possible to effectively filter out noise factors and obtain more accurate distance estimation.
- Real-World Case Studies: we performed the flight search test in two real-world test cases to verify the performance of the proposed survivor location tracking system. We operated fixed-wing drones in about 4 km × 4 km and 1 km × 1 km sites to search a survivor, and we compared the estimated position of the survivor with the actual position.

The rest of the paper is organized as follows: Section 2 introduces positioning and tracking systems and discusses problems and limitations of current disaster rescue platforms. Section 3 presents an architecture of the overall search platform and explains the detailed algorithm and implementation of the proposed smart search. In Section 4, we describe the experimental procedure and analyze the results for the performance evaluation of the proposed approach. Finally, Section 5 wraps up this paper with a discussion.

## 2. Related Works

### 2.1. Autonomous Path Planning

For completing an autonomous flight mission to a destination, planning with obstacle avoidance is the most basic and important process. There have been many research studies for efficient and effective path planning [4,5]. Several ML (Machine Learning) based approaches have been proposed for path planning. DQN (Deep Q Networks) [6,7,8] is one of the well known and widely used ML-based path planning algorithms. DQN is basically categorized as a reinforcement learning method [9,10,11], which learns how to make the best decision in the future through the process of performing an action and receiving a reward. Based on DQN, many researchers have developed various applications and extensions [12,13]. In addition, for realistic driving scenarios which require continuous actions, not discrete ones, DDPG (Deep Deterministic Policy Gradient) algorithm [14] adapts the ideas underlying the success of DQN to the continuous action domain. Many extended studies of DDPG have been developed for various applications [15,16,17,18].

The main limitation of the above methods is that these algorithms do not perform well, especially in new environments that differ significantly from the trained domain a priori. Compared to these conventional methods, agents in Value Iteration Networks (VIN) algorithms can learn a plan to reach a goal, even in a new environment [19,20,21]. Although VIN is also categorized as a reinforcement learning-based method such as DQN and DDPG, VIN has additionally embedded separate explicit reactive planning modules to express the policy. However, traditional VIN-based algorithms cannot cover a wide area. Thus, in this paper, we adopted a hierarchical VIN [22] method for path planning to cover a large target area such as our test fields (4 km × 4 km and 1 km × 1 km).

### 2.2. Localization Algorithm

A lot of effort has been put into accurate location tracking. These localization algorithms can be classified into anchor-based and anchor-free methods. In anchor-based algorithms, there are multiple anchor nodes with known positions obtained by GPS or other infrastructure. Using such information of anchors, locations of unknown nodes are estimated [23,24,25,26,27]. For example, an ad-hoc positioning system (APS) [23] is known to work well especially with the high connectivity between nodes. In addition, multilateration algorithms use least-squares estimation to compute positions and a Kalman filter [28] to avoid accumulating errors. Recently, many researchers have adopted ML-based (Machine Learning) methods to overcome NLOS (Non Line-Of-Sight) limitations [29,30] by combining Bluetooth and ultrasound signals and applying machine learning algorithms on the received signals.

As anchor-free localization methods, many researchers proposed mobile beacon methods [31,32,33,34]. Since localization error is reduced as the number of anchor nodes increases, several localization algorithms consider each of the dynamic locations of moving nodes as an individual virtual “anchor” [31,32]. Other anchor-free algorithms [33,34] select anchor points in RSSI-Range circles and track locations using the properties of the perpendicular bisectors of circles and chords.

However, the above methods are not suitable to be applied to our target scenario, where the target area is large and drones are moving fast for expedited rescue operation. To complement the limitations of the above anchor-based and/or anchor-free localization methods, we designed a genetic-based localization strategy. The GA (Genetic Algorithm) is known to provide a robust and global solution in many optimization problems, especially when the target problem is a multi-objective optimization in noisy environments. Since GPS and other infrastructures might not be available in our target scenarios, noise factors in the disaster rescue site can be increased. In addition, GA can search a population of candidate solutions in parallel and avoid local optimal solutions. Traditional methods, on the other hand, can get stuck in local solutions because they search from a single point. In our previous work [35], we first developed a genetic-based algorithm to obtain an accurate location by minimizing error factors of moving searching nodes as well as a target. However, when we applied this version of the genetic-based algorithm for real-world UAV based search systems, we found several issues. Hence, we re-built the genetic algorithm with which we can customize the system for considering flight environment and target area. We explain the detail of the genetic-based localization algorithm in Section 3.3.2.

## 3. Proposed Smart Search System

In this section, we introduce an overall architecture of the UAV-based smart search system and explain the details of each module of the proposed system.

### 3.1. Overall Architecture of the UAV-Based Smart Search System

**Figure 1:**
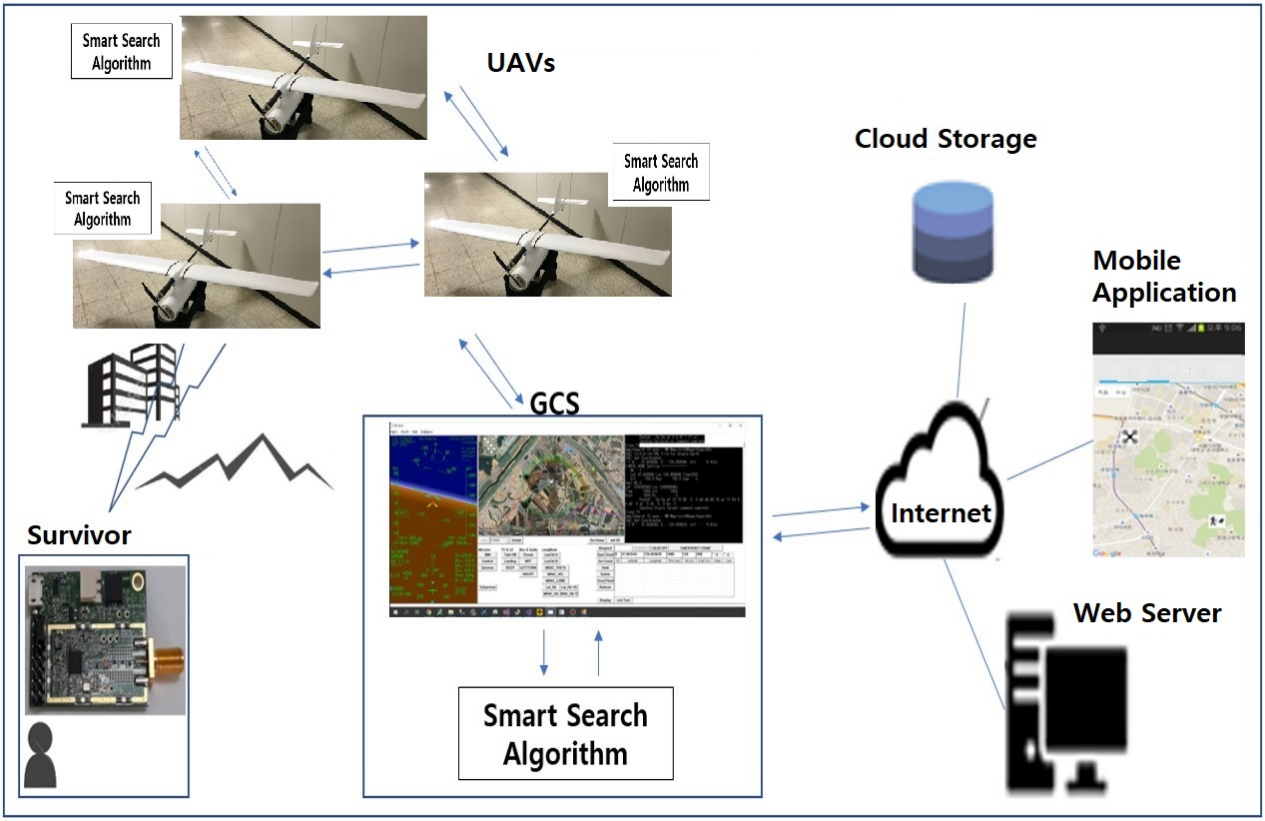
Overall smart search system for disaster rescue.

Figure 1 illustrates an overall system overview of the UAV-based location tracking system. A single or multiple UAV flies over the target disaster area and, when a signal is detected, it transmits information such as the drone’s latitude, longitude, RSSI, and ToA received from the survivor communication module to other UAVs and the GCS (Ground Control System) for data sharing. Based on the input data, the GCS and UAVs estimate the location of the survivor via the proposed Smart Search Algorithm. Specifically, the search algorithm estimates locations, updates the estimated locations of the survivor, and reduces the error between the estimated locations and the actual location. GCS is also uploading this information such as trajectories of UAVs and the estimated survivor’s location to its web server for further sharing with mobile applications.

In addition, GCS can store these data into cloud storage because the volume of these data is too large to keep in a local storage. All relevant information can be exchanged between UAVs via direct communication without going through a web server or shared with a web or smart device application via the server.

**Figure 2:**
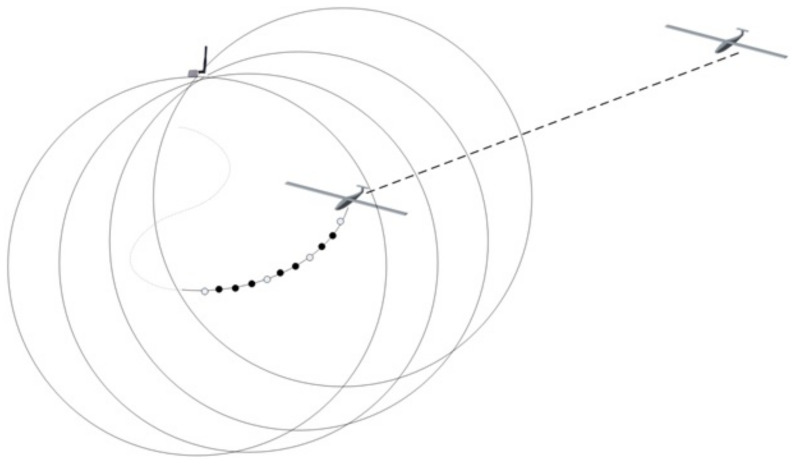
A sequence of estimated location during autonomous flight.

On the other hand, UAVs update their waypoints based on the estimated location of a survivor and fly toward the potential location. Figure 2 illustrates how the estimated position information indicated by the dots in this figure is continuously updated as a UAV approaches the target (The plain dots are waypoints for flight and the solid dots are temporary or local goals in flight trajectory towards the waypoints, which are generated by a trajectory builder inside UAVs).

### 3.2. Communication among UAVs and Ground Control System

Basically, we use a CSMA-based MAC layer protocol between two nodes [36]. Each agent in UAVs and GCS is considered as a node for communication. The parameters of a communication RF module on each node is summarized in Table 1. We have tried communication tests in a CSMA manner with varying the number of maximum re-transmissions and decided to allow four retransmissions for a higher success rate of communication. With using up to four retransmissions, the success rate of direct communication between two nodes is about 98%. We also construct a mesh network topology among multiple UAVs and GCS, and we adopt an OLSR (Optimized Link State Routing) [37,38] routing protocol over the mesh topology for exchanging localization-related data.

**Figure 3:**
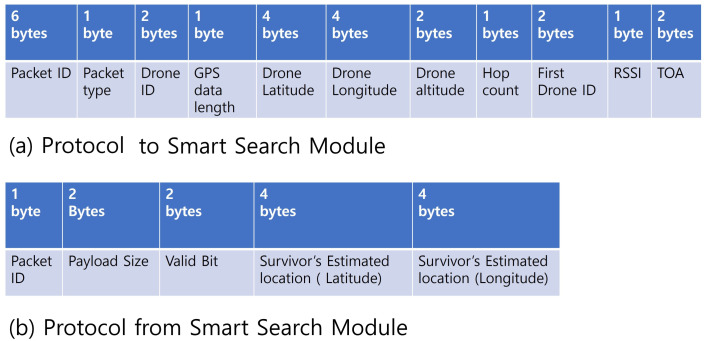
Communication Packets (**a**) to a Smart Search module and (**b**) from a Smart Search Module.

To exchange localization-related data among GCS and UAVs, we designed a packet in Figure 3a, which shows detailed fields which each packet consists of. Each UAV exchanges and shares data about a survivor and its own location data with other UAVs and GCS. Specifically, it sends packets containing drone ID to identify packets, types, and drones, as well as drone latitude, longitude, altitude, RSSI, and ToA data at the time of receiving the survivor’s signal.

Using this information, each UAV and GCS can launch its own Smart Search module to estimate a survivor’s location. For example, in the two test cases in the paper, the UAV and GCS exchange packets and each of them calculate the survivor’s location for itself. Note that the Smart Search module is embedded in each UAV and GCS.

The packets shown in Figure 3b are transmitted from a Smart Search module to a UAV or GCS agent. With this packet, the Smart Search process transfers the estimated location of the survivor, the ID indicating that it came from the Smart Search process, the size of the payload, and the value bits indicating whether the estimated location is valid or invalid. The latitude and longitude of the estimated location are represented by HEX. For the multi-byte data, it is represented by MSB first as well.

The communication procedure for searching a survivor is summarized as follows:

1. Each drone takes off and flies in a random direction until it catches signals from a survivor. We call this stage a random flight.
2. Once a radio signal with the survivor’s radio module is detected, the UAV shares and exchanges the information with other UAVs and GCS over the mesh network using OLSR routing protocols.
3. Each agent in UAVs and/or GCS launches a Smart Search Module and transfers the shared data about a survivor to the Smart Search Module.
4. The Smart Search module estimates the survivor’s location, if possible. If the obtained data are not enough for localization, then it sends back the packet with an invalid tag on.
5. If the Smart Search module sends valid location information of a survivor, then the GCS and UAVs switch their stage to a search mission for the survivor and generate an evasive path to the estimated survivor’s location.
6. UAVs autonomously fly to the waypoints avoiding obstacles towards the estimated location.
7. As an UAV gets close to the survivor, it gets more accurate signal information about it. It re-sends the information about a survivor to the Smart Search Algorithm and updates a survivor’s location (then go to Step 3).

**Figure 4:**
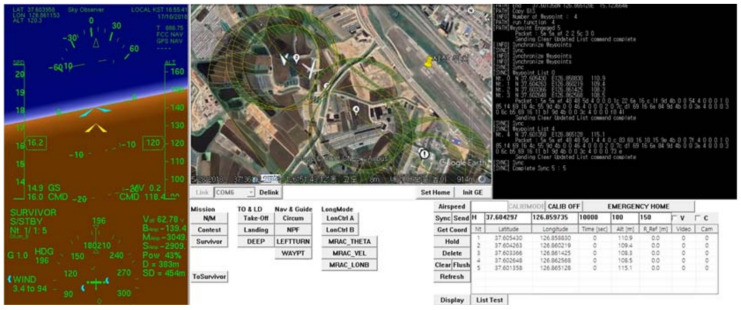
GCS (Ground Control System): the screenshot during the smart search flight.

In this system, GCS can check the drone’s latitude and longitude, altitude and inclination, wind direction, and wind speed during flight. It also links with Google Earth to determine the drone’s flight path and views the drone’s location on the map and the estimated location of the victim. Figure 4 shows the screenshot of our GCS during the smart search mission of UAVs. Thus, GCS with a human control can guide UAVs for more accurate and faster tracking by considering flight environment.

Note that the UAV of the proposed system is still able to estimate the location of the survivor by itself based on its own data even if the UAV is isolated and fails to communicate with other UAVs and GCS. Even if communication fails, it can reach the survivor by itself. However, the tracking procedure might be delayed or inaccurate because more data on survivors are better for genetic-based algorithms to calculate the location of survivors.

### 3.3. Smart Search Algorithm

We introduce the Smart Search Algorithm, which estimates the location of the victim based on the location of the drone, RSSI, and ToA values by considering flight environment as well. In our previous work [35], we first developed a genetic-based localization algorithm in a two-dimensional zone to obtain an accurate location by minimizing error factors of moving searching nodes as well as a target. However, when we applied this version of the genetic-based algorithm for real-world UAV based search systems, we found several issues. Hence, we re-build the genetic-based localization algorithm, with which we can customize it for considering flight environment and target area. The main differences are summarized as follows.

- This paper considers ToA as well as RSSI to estimate distance to a survivor because the target area of this paper is much bigger than a previous one. RSSI is more susceptible to noise over long distances and so we additionally consider ToA along with RSSI. Depending on how far UAVs are from the survivor, we adapt a reflection ratio of ToA and RSSI dynamically in this paper. Specifically, we estimate the position of the survivor by effectively combining RSSI and ToA information of radio signals that keep changing in real time during drone movements. The detailed discussion will be presented in Section 4.2.
- We re-designed a genetic-based algorithm by re-defining mutation and cross-over procedures. We also developed a new way to select individuals in the genetic algorithm as explained in Section 3.3.2.
- The proposed algorithm enables a UAV to fuse various localization data obtained from other UAVs by supporting multiple UAVs’ data considered to form a set of potential locations of the survivor.
- The input values required to execute this algorithm are the drone’s latitude, longitude, and altitude because we are dealing with a 3D zone in this paper.
- The proposed genetic-based Smart Search module is verified and finely customized to its flight environments.

#### 3.3.1. Distance Estimation

To accurately locate a survivor, we first need to measure the distance from a UAV to the victim. We, first of all, utilize RSSI (Received Signal Strength Indicator) information which can indicate the strength of the wireless signal. The higher the number, the stronger the signal is. Theoretically, RSSI values can give distance information between communication beacons [39]. However, RSSI values are affected by various environmental factors, such as the direction of the antenna, obstacles between beacons, or jamming propagation. An expression that translates the received signal strength through RSSI into distance is as follows when you develop a formula [39] that differs from the loss value from the transmission signal strength to a certain distance:

(1) $$d=10^{\left((Txpower-RSSI)/10n\right)}$$

In the equation, *d* is the distance, Txpower is the transmission power, and *n* is the indirect constant in the real world. For *n*, the value varies depending on the environment, but it is usually considered as 2 [39].

ToA, like RSSI, can estimate the distance between drones and survivors. ToA means the travel time of the radio signal from the transmitter to the receiver. The radio wave arrival time is estimated from the difference between the radio wave transmission time and the radio wave reception time at the signal source. Therefore, it is possible to estimate the distance between the drone and the survivor by multiplying the radio wave arrival time by the radio wave’s moving speed. Based on the data estimating the distance between the drone and the distressed using RSSI and ToA, it is possible to roughly find out the position of the survivor using triangulation.

**Figure 5:**
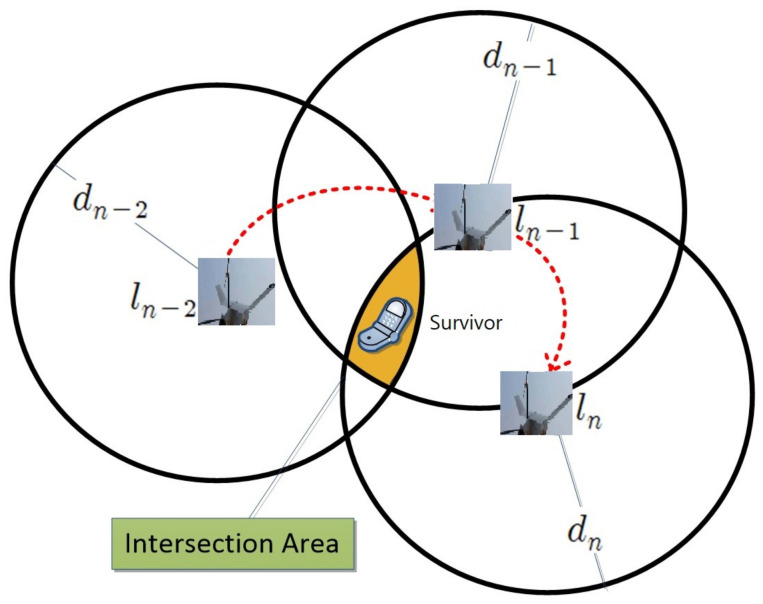
Triangulation method.

Triangulation is the most commonly used method for estimating the position of an object in two dimensions. At least three reference points are required to use triangulation. In this work, the position of the drone when it receives data from the survivor becomes the reference point. If the received signal strength is ideal, representing the converted distance based on the received RSSI value or ToA at each of the three reference points in one circle might have one point of intersection. The intersection point can be the estimated position of the survivor as shown in Figure 5.

To reduce the error for accurate localization, we developed a genetic-based localization algorithm as explained in the next section.

#### 3.3.2. Genetic-Based Localization Algorithm

The main idea for UAV-based location tracking systems is based on a genetic algorithm. The genetic algorithm mimics the evolution of life on Earth as it adapts to its environment over generations. In contrast to a baseline exhaustive search algorithm, which explores the number of possible cases and finds the best solution, we find a good solution based on the fitness value calculated by putting the years from generation to generation into the evaluation function.

The concepts of chromosome, gene, offspring, and goodness of fit are included to model genetic algorithms. Chromosomes in the genetic algorithm represent solutions. In the proposed location tracking system, we map each chromosome to each estimated location point for the survivor. The estimated location component, i.e., a chromosome *c*, is composed of latitude, longitude, and altitude. Progeny refers to chromosomes produced by crossing-over and mutation from the chromosomes of a parental generation. The fitness is a value that each chromosome has, and it is a value that expresses how well the chromosome is a suitable solution to the target problem. We explain the detail of the proposed genetic algorithm as follows.

Specifically, $n^{th}$ location, $l_{n}=\left(x_{n},y_{n},z_{n}\right)$ is shown in Figure 5 and $d_{n}$ is the distance from a survivor to the UAV calculated based on a combination of ToA or RSSI signals. The above notation in Figure 5 is assuming only one UAV in the system and each location of the same UAV is considered as different logical anchors. We extend it to support multiple UAVs and we use $l_{n}^{i}$ instead of $l_{n}$, i.e., $l_{n}^{i}$ means $n^{th}$ location of $i^{th}$ UAV. The distance between this UAV’s location and a survivor, $d_{n}^{i}$, is defined as follows:

(2) $$\begin{array}{c} d_{n}^{i}=\alpha \times d_{n}^{i}\left(RSSI\right)+\left(1-\alpha \right)\times d_{n}^{i}\left(ToA\right) \end{array}$$

where α=[0,1]. $d_{n}^{i}\left(RSSI\right)$ and $d_{n}^{i}\left(ToA\right)$ are the distance calculated by RSSI and ToA, respectively. The procedure and notations of the proposed GA-based localization algorithm is well illustrated in Algorithm 1 and Table 2. In the following, we explain the details of each function in this pseudo code.
**Algorithm 1 **Genetic-Based Localization Pseudo Code.1:**procedure** Proposed Algorithm2:     generation←0;3:     **while** !terminated **and** *generation* < Max_Gene. **do**4:         Collect Beacons();5:         **if** Collection fails **then** continue to line 3;6:         **end if**7:         Obtain Samples();8:         Form Population();9:         **if** rand [0, 1] > *crossrate* **then** Crossover();10:       **end if**11:       **if** rand [0, 1] > *mutationrate* **then** Mutation();12:       **end if**13:       Evaluate();14:       Terminated();15:       **if** terminated **then**16:             Unknown Node localized.17:             break;18:       **end if**19:       generation++;20:    **end while**21:**end procedure** 

**Figure 6:**
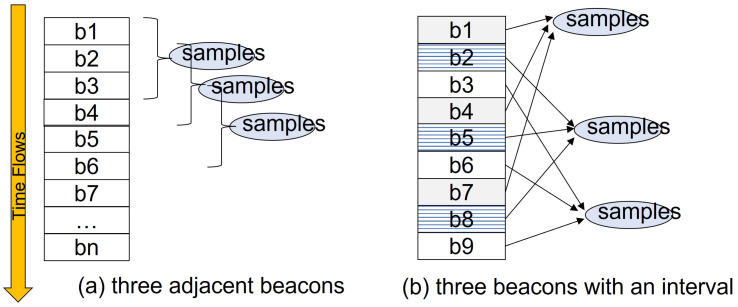
Two ways of selecting beacons in Collect_Beacons().

- Collect Beacons(): The Smart Search module in UAVs gathers its own beacons and also beacons from other UAVs, referred to as $b_{n}^{i}=\left(l_{n}^{i},d_{n}^{i}\right)$. Among these collected beacons, there are two different ways to select three beacons:
  -  three adjacent beacons from all UAVs (Figure 6a)
  -  three beacons from all UAVs with a certain time interval (Figure 6b)
  If the positions of the forming circles are close to each other, the width of the intersection section might be too large and the estimated positions could not be narrowed down. Thus, the proposed genetic-based algorithm uses the second method to select beacons so that selected circles can keep the proper distance to form an intersection area.
- Obtain Samples(): Smart Search modules draw three circles, each of which is correspondent to one of three beacons in the previous step. As shown in Figure 5, the three circles might create an intersection area instead of an exact single location point due to the distance errors calculated by RSSI and ToA. Therefore, the location should be estimated based on the intersection area, not a point of intersection. Then, the algorithm randomly extracts a certain number of sample points out of the intersection area and puts them into Samples. Note that, if the size of intersection area is way too large or too small, then we skip the genetic process and wait for the next beacons.
- Form Population(): The algorithm now selects a set of chromosomes, *c* out of Samples using fitness function. Fitness value of *c* is defined as follows:
  (3) $$\begin{array}{c} Fitness\left(c\right)=\sum_{b_{n}^{i}\in Selected_{B}eacons}\frac{1}{{\left(\mathrm{dist}\left(c,l_{n}^{i}\right)-d_{n}^{i}\right)}^{2}} \end{array}$$
  A fitness value for each chromosome is calculated based on the distance between the chromosome and the drone, and the radius of the circle data. In this work, we design a fitness function so that the closer the distance between the drone and the chromosome is to the distance estimated based on RSSI and TOA signals, the higher the fitness. The selected chromosomes form a population of a generation.
- Crossover() and Mutation(): It then undergoes inter-chromosome crossover and mutation, which constitute a generation with a certain probability. The following equations describe crossover process in the proposed algorithm in Algorithm 1:
  (4) $$\begin{array}{c} c_{a}=\left(x_{b},y_{b},z_{b}\right)\times \left(1-\alpha \right)+\left(x_{a},y_{a},z_{a}\right)\times \alpha \end{array}$$
  (5) $$\begin{array}{c} c_{b}=\left(x_{a},y_{a},z_{a}\right)\times \left(1-\alpha \right)+\left(x_{b},y_{b},z_{b}\right)\times \alpha \end{array}$$
  where $c_{a}=\left(x_{a},y_{a},z_{a}\right)$ and $c_{b}=\left(x_{b},y_{b},z_{b}\right)$ are selected chromosomes, and α is a random value in the range of [0, 1]. Then, we perform mutation with two offspring generated by crossover as follows:
  (6) $$\begin{array}{c} c_{a}=\left(x_{a},y_{a},z_{a}\right)\pm \left(\gamma_{x},\gamma_{y},\gamma_{z}\right) \end{array}$$
  where $\gamma_{x},\gamma_{y},\gamma_{z}$ are random numbers in the range of [0, minimum radius of three circles]. Such mutation and cross-over operations with randomness are performed to avoid local optimal solution.
- Termination(): the output offspring of above mutation and crossover operations are put into population and two worst-fitted chromosomes are removed from the population. This process is correspondent to one generation. If the best fitness value among the population is greater than a threshold or the number of generation is larger than a maximum generation threshold, then we terminate this process. If not, we repeat the above process.

### 3.4. Overall Procedure of Operations

**Figure 7:**
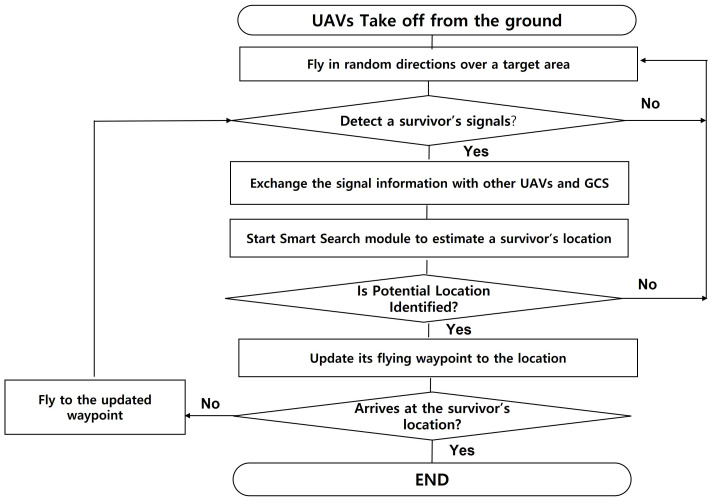
Overall procedure of operations.

The flowchart in Figure 7 illustrates the overall operation procedure of the proposed system. UAVs take off and fly in random directions until they catch signals from a survivor. Once a radio signal with the survivor’s radio module is detected, the UAV shares and exchanges the information with other UAVs and GCS. When UAVs and/or GCS collect enough information (i.e., beacons), then they launch their own Smart Search modules. The Smart Search module runs the proposed genetic-based localization algorithm to estimate the survivor’s location. If the potential location of a survivor is identified, even though it is not accurate yet, the UAV updates its waypoint for flight. If the UAV arrives at the identified location, it ends its mission and notifies it to GCS. If the survivor is still far from the current UAV’s location, then it flies towards the identified location and keeps collecting beacons. Based on the updated beacons, it re-estimates the potential location of a survivor and updates its waypoint again until it reaches the survivor.

## 4. Case Studies

This section presents results and analysis of the case studies conducted in the real world. In particular, we estimate the position of the survivor based on the data communicated with the survivor while operating the fixed-wing and rotary-wing drones, and we analyze the estimation error by comparing the estimated position with the actual position of the survivor.

### 4.1. Test Environment

**Figure 8:**
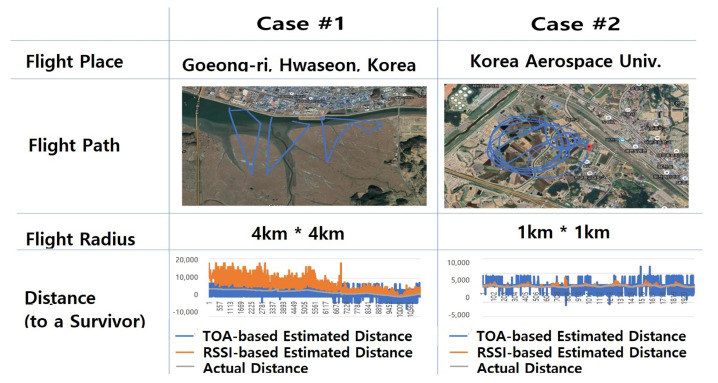
Two flight test case environments for verification of the proposed survivor location tracking system.

As shown in Figure 8, we performed a flight search test in two real-world test cases to verify the performance of the proposed survivor location tracking system. The first case was based on data obtained with a zigzag-type route from about 4 km × 4 km in Gojeong-ri, Hwaseong of South Korea. The second estimate was based on data obtained with a circling flight route within 1 km of the survivor in the Korea Aerospace University’s flight runway. In these two case fields, we examined features of signal-generation data (RSSI and ToA) obtained by operating drones to the distance and estimated the survivor’s location based on the data.

In both tests, we use one UAV and one survivor. However, theoretically, there is no limit of the number of drones as long as a routing protocol of a mesh network can support them and the target flight area is large enough to accommodate them. Note that we were able to localize a survivor even with the use of one UAV. If we use multiple UAVs, then we might be able to locate survivors more quickly and more accurately.

The UAV model used in both test cases is the Sky Observer [40], and the GCS runs on an Intel i5 CPU core with the Windows 10 operating system. The parameter values used in the proposed genetic localization algorithm are presented in Table 3.

### 4.2. Results: Distance Estimation

To localize the survivor, we need to accurately estimate the distance to the survivor. In this paper, we calculated the distance between the survivor and drones based on RSSI and ToA of signals. This section analyzes the estimation results tested in Case #1 and Case #2.

**Figure 9:**
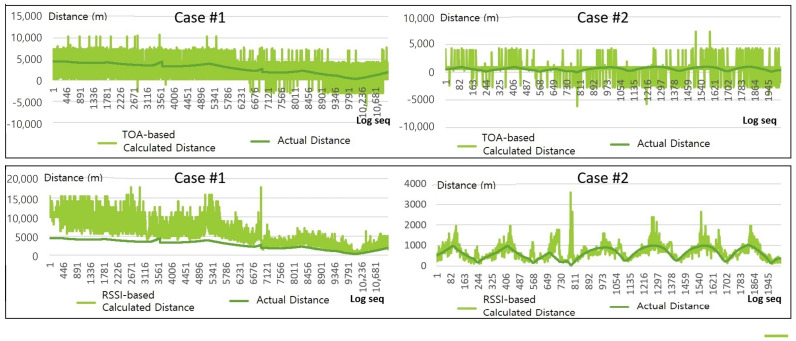
Estimates based on signals and actual distance graph between drones and survivors.

In Case #1, the survivor’s location is relatively far from drones’ departure position, i.e., about 4 km away as shown in Figure 8. The estimated distance based on the RSSI signal in the beginning of the flight search procedure is determined to be unstable showing severe fluctuation of the signal strength, represented as orange lines in the left bottom graph of Figure 8. However, as the drones moved closer to the survivor’s location within a radius of 1 km, the signal’s fluctuation gradually becomes decreased and localization errors were reduced. On the other hand, for ToA signals represented as blue lines in Figure 8, the error between the estimation and the actual distance is not much affected by the distance to the target. When the drone is far from the target, ToA-based estimation is relatively better than the RSSI-based one while, when the drone is near the target, the ToA-based one is worse. Graphs in Figure 9 are the detailed version of graphs in the bottom of Figure 8.

In Case #2, we also observed that the distance between the drone and the victim was relatively close, and that the RSSI signal showed no significant fluctuation, unlike the result in Case #1. As a consequence, RSSI-based distance estimation showed significantly reduced error. However, ToA-based distance estimation in Case #2 was still very unstable, which is not different from the one in Case #1.

This observation about RSSI-based estimation is trivial because the shorter the distance, the smaller the RSSI signal noise. To explain the reason for the above ToA results, we examined the experimental logs in more detail. Note that we converted ToA signals into distance using the following baseline equation:

(7) $$\begin{array}{c} Estimated\_distance=signal\_speed\times (ToA-system\_overhead) \end{array}$$

When calculating the ToA, there may be additional processing time consumed by the node (e.g., running the operating system and/or processing network traffic). To account for this system overhead, we reduce the total round trip time by the system overhead.

**Figure 10:**
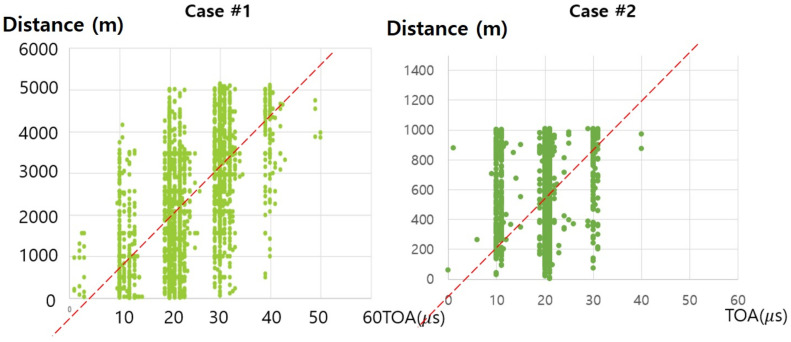
Distance between drones and survivors per ToA value graph.

Figure 10 shows a real distance between a drone and a survivor (i.e., *y*-axis) per each obtained ToA value (i.e., *x*-axis). Each dot corresponds to each measure. These graphs show that, even if the ToA values are the same, the corresponding values of distances do not converge but varies. This implies that the error of the ToA-based estimated distance would be large regardless of how far a drone is from the survivor. To ameliorate this problem, we used linear regression to obtain the “realistic” value of speed of ToA signals with minimal error in each case field. The value is expressed as a first-order straight line in each graph of Figure 10. Note that, even with the use of linear regression value, ToA-based estimation error would not be negligible. Similarly, in order to minimize the error of the RSSI signal-based distance estimation, this paper determines the value of a variable *n* in Equation (1) based on the measured data in each test case field.

**Figure 11:**
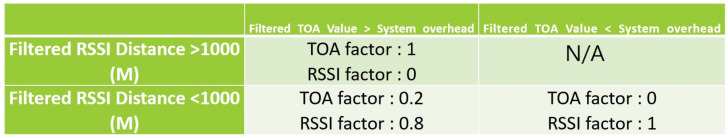
Reflection ratio of RSSI and ToA Signals.

Overall, we observe that errors of RSSI and ToA signals are related to the distance between a drone and a survivor. In particular, we discovered the tendency that RSSI-based distance measure is more accurate than ToA-based measure near a survivor while ToA-based shows better accuracy when a drone is far from a survivor. Thus, to increase the accuracy of the location estimation system, this paper adopts a “hybrid” of RSSI- and ToA-based methods depending on the estimated distance between a drone and a survivor. We separate the estimated distance values into two categories: above and below 1000 m as shown in Figure 11. Basically, if the estimated distance value was more than 1000 m, the RSSI value had a very large error and was difficult to be used as meaningful data. Therefore, distance estimates were made based on the filtered ToA values only when the distance is more than 1000 m. On the other hand, if the estimated distance is less than 1000 m, we increase the RSS reflection ratio gradually because we observe that the error of the RSSI value is reduced near a survivor. Note that we also exclude abnormal ToA-based signals from distance estimation procedure when the filtered ToA value is smaller than system overhead.

**Figure 12:**
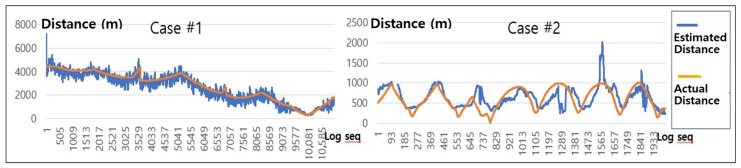
Estimated distance and actual distance between drones and survivors by the hybrid localization method.

Using the hybrid method of RSSI and ToA as shown in Figure 11, we track the location of a survivor in both Case #1 and Case #2. The results in Figure 12 compared the estimated distance from the above filtering process with the actual distance. For both Case #1 and Case #2, we found that the localization errors were significantly reduced compared to RSSI-only and ToA-only in Figure 9.

### 4.3. Results: Survivor Tracking

**Figure 13:**
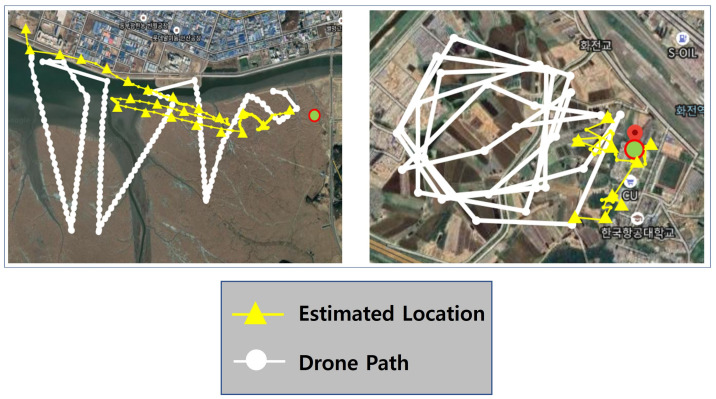
Estimated location of survivors.

In this section, we present a survivor tracking results obtained in two test fields. The paths shown in Figure 13 in yellow lines represent a sequence of estimated location of a survivor calculated as the proposed smart search procedure proceeds while the paths shown in white lines are the drone’s trajectory. The actual location of the survivor is marked with a red circle in both pictures in Figure 13.

For the first case in the left picture, the drone initially takes off at the left top corner in the picture, and the victim is located about 4 km away from the drone’s initial position. At the beginning, a drone has no idea about where a survivor is and so it randomly chooses a way point of flight. In this case study, the drone is flying in the southeast direction at the beginning as shown in the picture and flew in a zigzag manner for a random search of a survivor. During this flight, a drone captures a survivor’s signals and estimates a survivor’s direction even if the estimated location is not quite accurate. While it keeps gathering signals from a survivor, a drone can roughly guess the direction of the survivor and flies in that direction towards a survivor.

As shown in the picture, the initial estimate was not good because it was about 4 km away from the victim and there was not enough data for location estimation. For example, the first estimated position points to the upper left of the figure. Note that the direction to the survivor is right and thus the drone can approach near a survivor. Even if the first estimated location is very far from the actual survivor’s location, we can see that the estimated location is increasingly heading towards a survivor as a drone accumulates RSSI and ToA data from a survivor as it flies.

For the second case shown in the right picture of Figure 13, a drone takes off in the center of the picture and flies in a circle. A survivor is located in the right part of a picture as shown in a red dot. The initially estimated position is at the right bottom of a picture which is obviously not an accurate location. However, as the circulation flight progressed, it was confirmed that the sequence of estimated positions is getting closer to the actual position of the survivor. In this example flight, the final estimation is about 80 m away from the actual survivor’s location.

**Figure 14:**
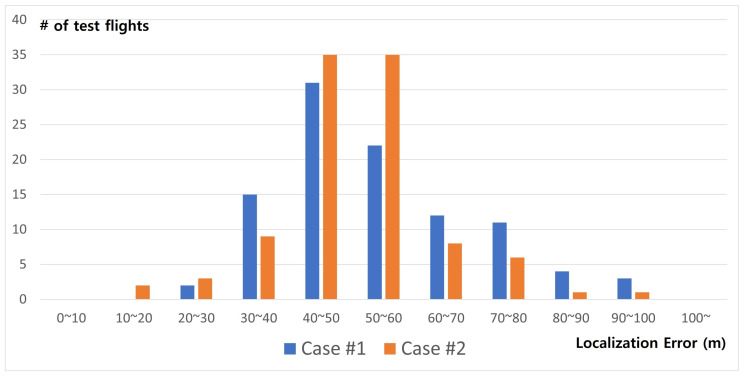
Minimum error frequency graph between distress and estimated location.

Just like the above two example flights, we tested the proposed Smart Search Program 100 times in Figure 14. These results show that, in most cases, localization error was about 40∼50 m. Note that the target area is very large about 4 km × 4 km in Case #1 and 1 km × 1 km Case #2, and we are using a high-speed fixed-wing plane, not a slow quad-copter. Since a fixed-wing plane cannot stop in the same position and it just has to hover in a large circle, we think the accuracy of estimation is limited in our study. To compensate for these limitations, it can be used not only with high-speed fixed-wing drones, but also with low-speed fixed-wing drones such as quad-copters. Once the location of the survivor is tracked in a small range, a more accurate location can be estimated using a low-speed fixed-wing drone.

## 5. Conclusions

This paper proposed a smart search system consisting of autonomous flying UAV, GCS, and smart search algorithms, and protocols. This system enables UAVs to perform autonomous flights while locating and approaching the distressed people even without direct control of the ground control server (GCS). When a UAV takes off, the first predicted position is not accurate and can be very far from the actual survivor’s position. As the drone flies, it accumulates RSSI and ToA data from survivors. As a result, the UAV gradually modifies its flight direction towards the survivor, resulting in a more accurate estimate of the location. For accurate localization, we also present a genetic-based search algorithm, which detects changes in the signal strength between distress and drones inside the search system. The proposed smart search system is customized to the disaster site environment to improve tracking accuracy. Specifically, by combining RSSI and ToA data in consideration of the flight environment, it is possible to effectively filter out noise factors and obtain more accurate distance estimation. Finally, we verified the whole proposed system in two real-world test fields, 4 × 4 km and 1 × 1 km, respectively, and found that it tracked down the survivor with about 20∼50 m errors out of 4 × 4 km and 1 × 1 km areas.

## Figures and Tables

**Table 1:** The parameters of a communication RF module.

Parameter/Measure	Type 1	Type 2
RF	Access-L	
Distance (km)	4.6	
PHY Data Rate (kbps)	9.6	
TX Power (dBm)	14 dBm (25 mW)	
RX LNA gain (db)	0	
Antenna	dipole 3.0 dBi	
# of Retransmissions	1	4
# of Bytes per packet	20	20
Success rate (%)	90	98
Mac Throughput (kbps) estimate	4	5
RSSI @RX (dBm)	−98	−98

**Table 2:** Notations in the proposed genetic-based localization algorithm.

Notations	Definitions
$l_{n}^{i}$	$n^{th}$ location of $i^{th}$ UAV $=(x_{n}^{i},y_{n}^{i},z_{n}^{i})$
$d_{n}$	the distance from a survivor to the UAV calculated
$b_{n}^{i}$	beacon composed of $\left(l_{n}^{i},d_{n}^{i}\right)$
Sample	randomly extracted survivor’s locations out of intersection area
*c*	chromosome correspondent to a survivor’s potential location
$dist(c,l_{n}^{i})$	distance between the chromosome and a UAV’s location

**Table 3:** Parameter values in the proposed genetic-based localization algorithm.

Parameters	Values
Population size	300
Crossover Probability	0.9
Mutation Probability	0.1
Threshold of MaxGeneration	300

## Abbreviations

The following abbreviations are used in this manuscript:

UAV	Unmanned Aerial Vehicle
GCS	Ground Control System
RSSI	Received Signal Strength Indicator
VIN	Value Iteration Network
DQN	Deep Q Networks
NLOS	Non Line-of-Sight
GA	Genetic Algorithm
ToA	Time of Arrival
CSMA	Carrier Sense Multiple Access
OLSR	Optimized Link State Routing
